# The Role of Flavonoids as a Cardioprotective Strategy against Doxorubicin-Induced Cardiotoxicity: A Review

**DOI:** 10.3390/molecules27041320

**Published:** 2022-02-15

**Authors:** Rony Abdi Syahputra, Urip Harahap, Aminah Dalimunthe, M. Pandapotan Nasution, Denny Satria

**Affiliations:** 1 Department of Pharmacology, Faculty of Pharmacy, Universitas Sumatera Utara, Medan 20155, Indonesia; aminah@usu.ac.id; 2 Department of Pharmaceutical Biology, Faculty of Pharmacy, Universitas Sumatera Utara, Medan 20155, Indonesia; pandapotan@usu.ac.id (M.P.N.); dennysatria@usu.ac.id (D.S.)

**Keywords:** doxorubicin, cardiotoxicity, cardioprotective, flavonoid

## Abstract

Doxorubicin is a widely used and promising anticancer drug; however, a severe dose-dependent cardiotoxicity hampers its therapeutic value. Doxorubicin may cause acute and chronic issues, depending on the duration of toxicity. In clinical practice, the accumulative toxic dose is up to 400 mg/m^2^ and increasing the dose will increase the probability of cardiac toxicity. Several molecular mechanisms underlying the pathogenesis of doxorubicin cardiotoxicity have been proposed, including oxidative stress, topoisomerase beta II inhibition, mitochondrial dysfunction, Ca^2+^ homeostasis dysregulation, intracellular iron accumulation, ensuing cell death (apoptosis and necrosis), autophagy, and myofibrillar disarray and loss. Natural products including flavonoids have been widely studied both in cell, animal, and human models which proves that flavonoids alleviate cardiac toxicity caused by doxorubicin. This review comprehensively summarizes cardioprotective activity flavonoids including quercetin, luteolin, rutin, apigenin, naringenin, and hesperidin against doxorubicin, both in in vitro and in vivo models.

## 1. Introduction

Doxorubicin is a part of the anthracycline group of chemotherapy, one of the most widely used and efficacious methods for treating hematological malignancies, solid tumors, and lymphoma [1]. The essential mechanism of doxorubicin involves generating oxidator and inhibiting topoisomerase II in cancer cells, although, on the other hand, it is toxic to several organs, including the heart [2]. Therefore, a severe dose-dependent cardiotoxicity hampers its therapeutic value. Based on the administration duration of doxorubicin, its cardiotoxicity is divided into acute and chronic toxicity, with acute toxicity occurring during the early administration of doxorubicin (within 2–3 days), in approximately 11% of incidences. Clinical manifestations of acute toxicity are hypotension, tachycardia and various arrhythmias, pericarditis, or myocarditis, but these are reversible with appropriate treatment [3,4]. Chronic toxicity occurs after 30 days of the administration of doxorubicin, but the percentage of toxicity is lesser than acute toxicity—about 1.7%. This can lead to left ventricular dysfunction that irreversibly evolves toward congestive heart failure [5].

Cardiotoxicity due to doxorubicin depends on the dose administration: normally a dose below 400 mg/m^2^ is less toxic. If the dose exceeds more than 400 mg/m^2^, it will increase the likelihood of toxicity occurrence [6]. In addition, medical history and age also influence the risk of cardiotoxicity. A history of diabetic or cardiovascular disease such as hypertension, hyperlipidemia, or atherosclerosis develops these complications [7]. Moreover, age >65 and >4 years old are the most vulnerable [8]. Tobacco use, poor nutrition, or being physically inactive also have a part in developing the risk of cardiotoxicity [9].

Several molecular mechanisms underlying the pathogenesis of doxorubicin cardiotoxicity have been proposed, including oxidative stress [10], topoisomerase II inhibition [11], mitochondrial dysfunction [12], Ca^2+^ homeostasis dysregulation [13], intracellular iron accumulation [14], ensuing cell death (apoptosis and necrosis) [15], autophagy [16], and myofibrillar disarray and loss [17].

Nowadays, preventive strategies have been delivered to reduce and prevent cardiotoxicity development. These are limited doxorubicin dose [18], liposomal formulation doxorubicin [19], and the co-administration of dexrazoxane with one of the cardioprotective FDA-approved drugs for preventing doxorubicin-induced cardiotoxicity. Up-to-date mechanisms of dexrazoxane are iron chelator, diminishing oxidative stress, and directly competing with topoisomerase II in the nucleus [20]. The early detection of cardiac biomarkers will be beneficial for patients. Cardiac biomarkers use for analysis such as cardiac troponin T (cTnT), brain natriuretic peptide (BNP), atrial natriuretic peptide (ANP), and c-reactive protein (CRP) which are known increase in cardiotoxic patients [21].

Natural products have traditionally been used by humans for food consumption, nutrition, and preventive measures and treatments for several diseases [22]. Flavonoid derived-plants mainly explored and investigated for cardioprotective measures against doxorubicin-induced cardiotoxicity [23]. Flavonoids are divided into the following classes: flavones, flavonols, flavanones, flavanonols, flavanols or catechins, anthocyanins, and chalcones. Each subclass of flavonids has been widely tested on in vitro and in vivo models of cardiotoxicity-induced doxorubicin [24]. Flavonoid has pharmacological effects such as antioxidant, anti-inflammatory, anti-apoptosis, anti-calcium overload, and iron scavenging properties [25]. The flavonoid divided into subclasses quercetin, rutin, apigenin, and luteolin have been researched the most in in vivo and in vitro models against doxorubicin. Particularly, the mechanism of protective activity is clear and well known; subsequently, increases in the expression of nrf-2, SOD, GSH, and HO-1 inhibit the expression of pro-apoptosis protein cytochrome c, Bax, caspase 3, caspase 7, caspase 9, and inhibit the expression of pro-inflammatory protein TNF, IL-1, IL-6, and Nf-kB [26,27,28,29,30]. Therefore, this comprehensive review provides a summary of the cardioprotective mechanism of flavonoids against cardiotoxicity induced by doxorubicin.

## 2. Doxorubicin Mechanism of Toxicity

### 2.1. Doxorubicin Generates Reactive Oxygen Species (ROS)

Doxorubicin is a well-known potent generator of ROS both in cytosolic and mitochondria [31]. The metabolite of doxorubicin is doxorubicin-semiquinone, which rapidly oxidizes oxygen (O_2_) converted into superoxide anion radical (O_2_^−^). Unfortunately, this O_2_^−^ is highly reactive with NO, which can produce peroxynitrite (ONOO^−^). The accumulation of ROS is normally cleared by endogenous antioxidants such as SOD, or exogenous antioxidants such as flavonoids that produce H_2_O_2_; however, the existence of iron (Fe^2+^) can directly convert H_2_O_2_ into hydroxyl radical (OH), which is a known Fenton reaction [32,33,34,35]. Moreover, in the case of high cumulative doses of doxorubicin, ROS production is also extremely high, which might cause the degradation of lipids in the membrane, decrease the ATP, induce the opening of MPTP, and sensitize the ryanodine receptor that induces the excessive release of calcium into the cytosol. All these ROS effects lead to the apoptosis of cardiomyocytes [36,37,38]. The supplementation of antioxidants will beneficially reduce the ROS overproduction of doxorubicin. Most of the reviewed research confirmed that flavonoids reduce the production of ROS, both in in vivo and in vitro models, but the mechanism remains unclear and needs to be elucidated [39]

### 2.2. Mitochondria Injury

Mitochondria is the source of energy for cardiomyocytes, uniquely, with mitochondria levels 60% higher in cardiomyocytes than in the rest of the body’s cells [40]. Hence, the volume of cardiomyocytes consists of 30–40% mitochondria [41]. As mentioned earlier, ROS production in the mitochondria is assumed to be the cause of mitochondria injury [42,43,44]. Doxorubicin has a strong binding affinity to cardiolipin in the inner membrane of mitochondria, that may directly cause the disturbance of the electron transport chain (ETC), which cause increased the ROS dan RNS [45,46,47]. Hence, the pro-oxidant causes opening pore mitochondria resulted release of cytochrome c that initiates apoptosis by activating caspase 3. Furthermore, this mechanism also causes mitochondria swelling, which leads to necrosis and rupture of the outer membrane of mitochondria. Both apoptosis and necrosis primarily cause the death of cardiomyocytes mostly mediated by ROS [48,49,50].

### 2.3. Topoisomerase 2β (TOP2β)

The main mechanism of doxorubicin as an anticancer agent is the binding to topoisomerase in the nucleus of cancer cells [51,52,53]. Topoisomerase has two isoforms which include 2α (TOP2α), and 2β (TOP2β) topoisomerase. In the tumor cell, doxorubicin binds into TOP2α, resulting in DNA degradation and cell death. Unfortunately, the toxicity of doxorubicin also binds to TOP2β, which is dominantly expressed in the adult cardiomyocytes [51]. These complex doxorubicin-DNA-TOP2β cause double-strand DNA breakage, which causes apoptosis [54,55,56]. Interestingly, doxorubicin seems more susceptible to binding to TOP2β in cancer patients. The accumulation of doxorubicin in cancer patients increases the doxorubicin-induced cardiotoxicity. Primarily, the maintenance of doxorubicin dosage in patients is the most important measure in preventing cardiotoxicity. In relation to the nucleus stress due to doxorubicin, the translocation of erythroid-2-related factor (Nrf2) to the nucleus will be provided by the Keap1-Nrf2 complex, which produces OH-1 as a protector [57,58,59]. Meanwhile, the stress condition activates c-Jun N-terminal kinases (JNKs) and p38-MAPKs by cellular oxidative stress, that also correlate with cardiac pathophysiology and apoptotic cell death [60,61,62].

### 2.4. Calcium Homeostasis Dysregulation

Regulating the level of Ca^2+^ levels in the cell are essential for maintaining calcium homeostasis. However, doxorubicin increases the level of intracellular Ca^2+^ [63,64], and it downregulates the expression level of SERCA2a, leading to a decrease in Ca^2+^ uptake [65,66,67]. SERCA2a plays a vital role in restoring the excessive amount of Ca^2+^ in the cytosol, the uncontrolled level of Ca^2+^ in cells causes the impairment of contractile cardiac muscles [68]. Furthermore, doxorubicin also inhibits the Na^+^/Ca^2+^ exchanger [69], and the increase in doxorubicin levels also causes an increase in the expression of the ryanodine receptor channels, which leads to the massive release of Ca^2+^ [70,71]. Interestingly, SERCA is inhibited by the ROS-mediated S-oxidation of the conserved Cys 674 along with increasing RyR2 [72]. The mitochondria absorb a large portion of the released calcium, and the receptor is known to be sensitive to oxidation due to presence of many thiols [73]. Overloading the mitochondria with calcium can result in mitochondrial malfunction and the induction of a cascade of pro-apoptotic events [74]. Furthermore, doxorubicin causes Ca^2+^-dependent Ca^2+^/calmodulin-dependent protein kinase II (CaMKII) activation as the result of promoting apoptosis [75].

### 2.5. Cardiac Biomarkers Injury

**Figure 1:**
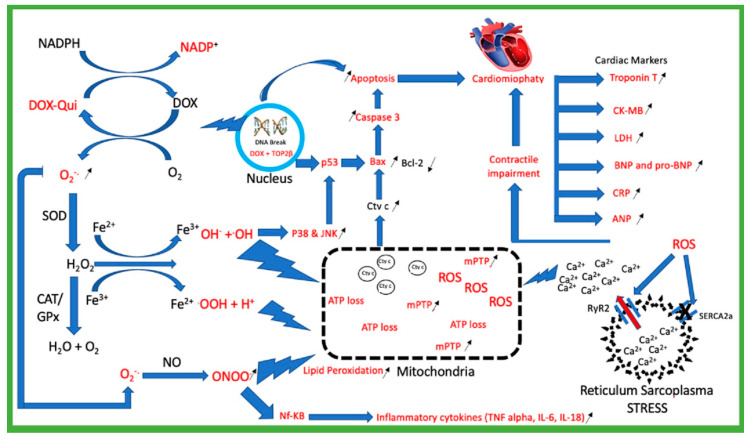
Doxorubicin mechanism of cardiotoxicity is doxorubicin converted into doxorubicin-semiquinone by transferring an electron from NADPH, while it changes into NADP+ continuously. However, an electron is transferred from O_2_ to doxorubicin-semiquinone, thereby creating O_2_^−^ (superoxide radical) neutralized by SOD into H_2_O_2_ which can be converted into H2O + O_2_. In cardiac toxicity events, the SOD and CAT leaves are down; hence, H_2_O_2_ is converted into OH^−^ and *OOH (hydroperoxyl radical) by the Fenton reaction. The superoxide radical is highly active, such that it directly damages the cell membrane, specifically the mitochondria membrane, which causes the increase in lipid peroxidation, and the membrane permeability of mitochondria also causes ATP loss. On the other hand, the superoxide radical also triggers the stimulation of protein P38 and JNK which activates protein p53 and increases caspase 3 activity. Furthermore, cytochrome c was released and activated the Bax (pro-apoptosis protein) which stimulates the activation of caspase 3 activity which increases apoptosis events. The Dox mechanism of action binding into topoisomerase 2β breaks the DNA that causes apoptosis of the cell. Additionally, ROS directly damages the reticulum sarcoplasm that causes the elevation of Ca^2+^ into the cytosol and increases the contractile, thereby causing contractile impairment. The accumulation of apoptosis in cardiac cells and contractile impairment leads to cardiomyopathy and the release of cardiac biomarkers such as Troponin T, CK-MB, LDH, BNP, NT-pro-BNP, ANP, and CRP.

Early detection of cardiac injury is beneficial to the patient in preventing the incidence of cardiotoxicity by doxorubicin. Many cardiac biomarkers have been used to predict a cardiotoxic event, one of which includes AST as the first biomarker to assess the cardiac injury. However, the limitation of this is that the release of AST occurs not only in the cardiac cell, but also in liver injury [76,77,78]. Moreover, LDH has been used as a biomarker for cardiac enzymes in the past; usually, the increase in the level of LDH is detected after 24–72 h of cardiac injuries [79]. Meanwhile, in terms of acute myocardial injury, CK-MB is more sensitive compared to LDH, and it is becoming an essential cardiac biomarker of injury [80]. CK-MB levels are higher in cardiac muscle rather than in skeletal muscle, which is primarily made up of CK-MM [81]. Presently, AST, LDH, and CK-MB are no longer recommended as early detectors of cardiac injury, but Troponin is used as an early detector (gold standard) instead [82]. Furthermore, Troponin is divided into three isoforms, namely the troponin T complex of the actin filament, the troponin c complex of Ca binding, and the troponin I complex of the myosin head [83,84,85]. BNP is an essential marker in heart failure, which is produced by the left ventricle when there is a myocardial stretch. The early detection of BNP is vital to prevent and assess future treatment [86,87,88]. The chronic cardiac toxicity of doxorubicin causes heart failure and particularly reduces fraction ejection. BNP affects increases in vasodilation, diuresis, and natriuresis. As a result, the American Heart Association recommends BNP and NT-proBNP as a biomarkers of heart failure [89,90]. C-reactive proteins (CRP), which are recognized as inflammatory markers, are potentially used to predict the adverse incidence of cardiac injury [91]. In many studies, both in vivo and vitro models of flavonoid-attenuated doxorubicin-cardiotoxicity have used AST, LDH, CK-MB, Troponin, BNP, and CRP as markers to predict the potency of flavonoids (quercetin, rutin luteolin, and apigenin) and emphasize the injury caused by doxorubicin. It has been acknowledged that these biomarkers are continuously used for the analysis of cardiac toxicity, but a more relevant study is needed to conclude on the biomarker that should be used to predict toxicity. Figure 1 shows the mechanism of doxorubicin increasing ROS, mitochondrial dysfunction, ER stress, DNA break, apoptosis, and cardiac markers.

## 3. Flavonoid

**Figure 2:**
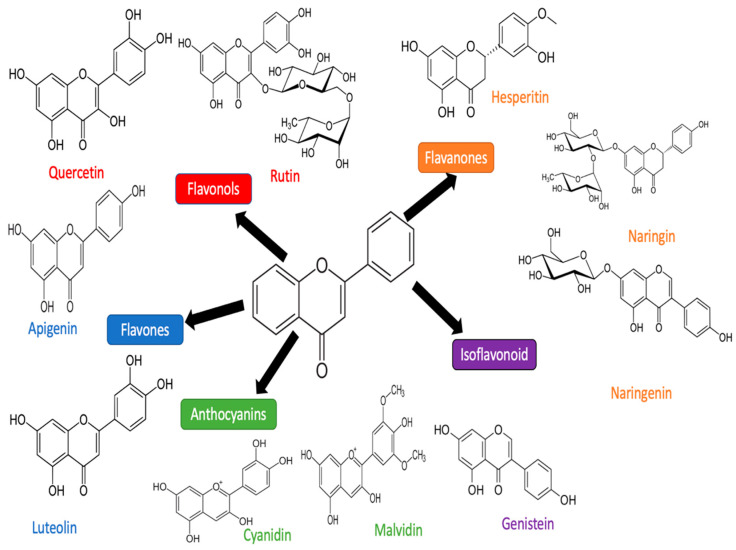
Flavonoid subclass structures (the same color represents the same subclasses of flavonoids).

Flavonoids are an essential group of a natural compounds, generally discovered in many plants [92]. Particularly, their pharmacological activities have been tested intensively in animal models, cell models, and human trials [93,94,95]. Some of them found the inhibition of Nf-KB [96], scavenging radicals [97], antiplatelets [98], anti-thrombotic [99], angiotensin-converting enzyme inhibitors [100], anti-carcinogenic [101,102], anti-calcium overload +, lipoxygenase inhibitors [103], etc. Flavonoids are categorized into some subclasses depending on the C ring and B ring, as well as unsaturation and the oxidation of the C ring. These are flavonols (quercetin, rutin, myristine, morin, kaempferol), flavones (apigenin, luteolin), anthocyanin (cyanidin, malvidin), flavanones (hesperetin, naringin, naringenin) and isoflavonoids (genistin, genistein) [104]. The flavonoid structures are shown in Figure 2.

### 3.1. Luteolin

Luteolin (3,4,5,7-tetrahydroxy flavone) is discovered in many natural resources, such as vegetables and fruits that are used daily in human life [105]. Furthermore, it has been tested in many pharmacological activities, including anticancer, antidiabetic, anti cholesterol, and cardioprotective measures against doxorubicin [106,107]. Moreover, it is known to inhibit the carbonyl reductase 3 for the conversion of doxorubicin into doxorubicinol [108,109]. In Chinese traditional medicine, plants rich in luteolin are used to treat inflammation, hypertension, and to increase the luteolin plays a vital role as an anticancer agent in multiple mechanisms, such as the suppression of kinase, the regulation of cells, and apoptosis. It has shown many positive effects regarding the multiple cardioprotective effects against ischemia/reperfusion, heart failure, and atherosclerosis [110,111,112]. The luteolin experimental study was conducted in both in vitro and in vivo models against doxorubicin-induced cardiotoxicity. Moreover, a study conducted by [113] reported that rats which were administered a cumulative dose of doxorubicin at 16 mg/kg, as well as luteolin of 50 and 100 mg/kg, for the luteolin groups, showed that both doses of 50 and 100 mg/kg attenuated the toxicity of doxorubicin. The cardiac biomarkers such as troponin T, BNP, and LDH in doxorubicin in the co-treatment luteolin group were significantly reduced. Meanwhile, in the other groups, only the rats which were administered doxorubicin significantly increased. Furthermore, luteolin increases the expression of phlpp1 and p-Akt protein. phlpp1 has been known to regulate the AKT protein to increase cell survival, which minimizes the apoptosis caused by doxorubicin. Interestingly, a similar result also reported that in the pretreatment of luteolin 10 and 20 µM on H9c2 induced by DOX, the 10 µM showed a depletion of ROS production. On the other hand, the H9c2 cell that was only given DOX significantly increased the ROS level, while the pretreatment of luteolin increased the expression of PTEN, and decreased ERK, AKT, and mTOR [114]. Recently, many studies have reported that mitochondria are the main target of DOX toxicity and have led to the opening of the mitochondrial permeability transition pore (mPTP) [115]. The PI3K/Akt and ERK play an essential role in cell proliferation, apoptosis, and migration of the cells [116]. Another study implied that DOX activated several downstream pathways, including PI3K/Akt, while the main role of luteolin is to block the phosphorylation of PI3K, which causes the decrease in ERK, AKT, and mTOR [117]. In an in vivo study, CK-MB, LDH, and some specific cardiac biomarkers including BNP, Troponin T, and CRP were used to assess cardiotoxicity rate [118]. An agreement study by Syahputra showed that rats induced by doxorubicin significantly increased their BNP and Troponin T levels [119]. In some studies, the antioxidant was widely determined by the abundance of ROS production. SOD plays the main role in the neutralization of O-, which is radically active in H_2_O_2_, and which is less toxic [120]. The previous study stated that doxorubicin reduced the SOD levels while pretreatment of luteolin increased the SOD levels [117]. Luteolin- 7-*O*-β-D-glucopyranoside isolated from Dracocephalum tanguticum can reduce the production of CK and LDH and inhibit the increase in ROS expression on H9c2-treated Dox [121]. Interestingly, luteolin has anti-calcium overload qualities, whereby Ca^2+^ plays an essential role in the contraction and relaxation of the cardiac muscle; therefore, it is important to maintain the level of Ca^2+^ level, since its imbalance could lead to the loss of cardiac function. SERCA2a plays an important role in maintaining the reuptake of this Ca^2+^. This luteolin significantly increased the SERCA2a expression in rats with an injured myocardium, which prevented contractile impairment [122]. Therefore, this study conducted proper documentation of the contribution of luteolin against doxorubicin-induced cardiotoxicity. Table 1 completely shows the study design and the doses administered for both luteolin and doxorubicin, and the durations and parameters.

### 3.2. Quercetin

Quercetin is a flavonol group that is generally found in many plants, such as berries, onions, green hot paper, apples, pears, spinach, etc. [123]. Furthermore, its daily intake in humans is estimated to be 20–50 mg [124]. This promising natural compound has been widely tested for numerous pharmacological activities, including anticancer, antidiabetic, anti-analgesic, and anti-inflammatory properties, and as a cardioprotective it encounters multiple causes such as doxorubicin-induced cardiotoxicity, ischemia/reperfusion injury, and diabetic cardiomyopathy [125,126]. Quercetin acts on several upstream and downstream signaling pathways of the cells such as cardiomyocytes, which are beneficial for cell survival. Quercetin, which downregulates the protein of ERK and MAP kinase on cardiac cell injury [127] was tested on H9c2 cells; the results showed that upregulating the expression of the Bmi-1 protein played the main role in ROS generation and mitochondrial function. Bmi-1 modulated the antioxidant defenses by suppressing the p53 pro-oxidant protein [128]. Furthermore, the H9c2 cell treated with quercetin showed a significantly reduced apoptotic effect, while the H9c2 cell that was only administered doxorubicin significantly increased the apoptotic cell. Moreover, in the in vivo models, mice treated with 20 mg/kg of doxorubicin alone significantly reduced in heart weight and heart-to-body weight ratio. The results of this study showed an increase in creatinine kinase (CK) and LDH as cardiac biomarkers in mice treated with only doxorubicin. Meanwhile, the mice treated with 100 mg/kg of quercetin during pretreatment significantly reduced the cardiac biomarkers [128]. The results clearly showed that quercetin counters the production of oxidative stress by doxorubicin. The simultaneous administration of quercetin with resveratrol on H9c2 cells showed a significant reduction in ROS, AST, ALT, and CK [127]. Interestingly, pre-treatment of quercetin with H9c2 elevated the expression of protein 14-3-3γ which is involved in the protection of myocardial injury. Therefore, pretreatment of quercetin suppressed caspase-3 activity, while the H9c2 cell which was administered with doxorubicin alone inclined the caspase-3 activity. The cell pretreatment of quercetin prevented the opening of mPTP, while in the Dox alone, mPTP was high such that it stimulated the swelling of mitochondria and the excessive release of ROS [129]. In agreement with a study reported by [130], the combination of 80 mg/kg quercetin with sitagliptin on a rat induced with a doxorubicin accumulative dose of 18 mg/kg showed a significant reduction of cardiac biomarkers LDH, CK, Troponin, and CRP. Meanwhile, the doxorubicin group showed a significant trend in these parameters. The combination of sitagliptin and quercetin was more potent compared to sitagliptin and quercetin alone against doxorubicin, and multiple mechanisms including antioxidant, anti-inflammatory, and lipid-lowering effects contributed against doxorubicin [130]. In correlation with the Asma study on an in vivo model, the co-administration of a quercetin dose of 10 mg/kg with losartan of 0.7 mg/kg, on rats induced with doxorubicin of 15 mg/kg, inclined with the myocardial antioxidant enzymes such as SOD and CAT, while the marker of oxidative stress MDA declined [131]. Moreover, the TNF alpha, which is already known as a pro-inflammatory cytokine that stimulates ROS, increased in doxorubicin alone. Interestingly, quercetin attenuated the TNF alpha and the Nuclear Factor-Kappa B (NF-κB). This inhibition was mediated by the antioxidant ability of quercetin. The combination of losartan and quercetin showed a better correlation against the toxicity of doxorubicin than losartan and quercetin alone, because of the synergetic effect [132]. An in vivo study stated that quercetin 2 mg/kg/day for 7 days attenuated the cardiac toxicity caused by DOX, which significantly changed the cardiac biomarkers and significantly improved cardiac histology [133].

### 3.3. Apigenin

Flavonoids are natural compounds in almost all plants tissue. One of them includes apigenin, which belongs to the sub-classes of flavone [134]. Apigenin has several interesting pharmacological activities which include antioxidant, inflammation, autoimmune, neurodegenerative, and antidiabetic effects, etc. [135]. Furthermore, it upregulates the cell signaling pathway PI3K/Akt and downregulates NF-κB, as well as reduces COX-2 expression. Interestingly, apigenin has well documented elevated antioxidant enzymes such as SOD, Catalase, and Glutathione for encountering cellular oxidants (Sahu et al., 2019). Interleukin 6 (IL-6) and the TNF alpha, which are known as pro-inflammatory cytokines, are attenuated by apigenin. In an antidiabetic animal model, apigenin regulated hyperglycemia and neutralized the reactive oxygen species (ROS) [136]. Moreover, it played a vital role in encountering cardiac remodeling, cardiac apoptosis, and toxicity due to doxorubicin [136]. Table 1 below shows a few reports on apigenin against doxorubicin. Apigenin is a common compound discovered in countless plants, and it has strong antioxidant activity. Some studies were conducted on behalf of the cardioprotective activity in both animal and cardiac cell experiments. A study conducted by Zara et al. showed that apigenin increased the body weight and heart body weight relative ratio in rats treated with doxorubicin. Hence, the ejection fraction was significantly improved, and there was no difference with normal rat groups. In addition, the percentage of fibrosis on the cardiac cell was decreased compared to doxorubicin alone [137]. Cardiac biomarker injuries, such as CK-MB, LDH, and troponin T, were decreased in the apigenin + doxorubicin group. The expression of anti-apoptotic protein Bcl was decreased, and the expression of pro-apoptotic caspase 3 and Bax was also decreased [137]. In agreement with the previous study, apigenin significantly reduced myocardial enzymes AST, LDH, and CK [138]. In addition, mitochondrial dysfunction has been recently highlighted as a major incense in rats treated with doxorubicin. Interestingly, the ratio of Bax/bcl2 was increased in the doxorubicin treatment group, although, on the other hand, the apigenin protein pro-apoptotic was reversed. Furthermore, apigenin also activated the PI3K/Akt/mTOR pathway in rats treated with doxorubicin, which increased apoptosis. Apigenin of 75 mg/kg leads to the activation of peroxisome proliferator-activated receptor-γ (PPAR-γ) on the rat-induced myocardial infraction, reducing the CK-MB and LDH, as well as reducing the expression of Bax. The stimulation of PPAR-γ decreased in size and inflammation [136]. A similar study conducted by [139] stated that apigenin attenuated myocardial/reperfusion injury by significantly reducing TNF alpha, phospho-IkB-, NF-κB, and ICAM-1. Interestingly, apigenin depleted the inflammatory biomarker of COX-2 and iNOS expression on rats.

### 3.4. Rutin

Rutin, or quercetin-3-O-rutinoside, is one of the flavonoid compounds which belongs to the subclasses of flavanol [140]. Furthermore, more than 60 plant species which include *Vernonia amygdalina* from the family Asteraceae contain rutin [141]. As with many other compounds of flavonoids, a major problem is poor bioavailability due to low solubility, and unstable and poor permeability [142]. Rutin is pharmacologically known to attenuate cardiac remodeling by blocking some cellular signaling pathways and ROS [143]. Doxorubicin is known to cause dilation of the left ventricle, leading to cardiac impartment, specifically reducing the ejection fraction. In addition, it generates ROS, apoptosis, lipid peroxidation, and inflammatory response, while endogenous antioxidant SOD, GSH, Catalase are reduced [144]. Rutin has been tested on several in vivo and in vitro models against doxorubicin, and the data is well documented in Table 1. Rutin has significantly shown a decrease in ROS and apoptosis in H9c2 cells treated with doxorubicin. Meanwhile, groups that were administered with doxorubicin only showed a significant increase in ROS and apoptosis [118]. A previous study stated that the activation of ROS-dependent p38 MAPK and the deactivation of ERK signaling pathway led to myocardial apoptosis by doxorubicin [118]. Interestingly, the existence of rutin in the H9c2 cardiomyoblast cell protects the activation of the p38 MAPK signaling pathway [118]. Moreover, the expression of pro-apoptotic proteins caspase 3, caspase 7, and caspase 9 in H9c2 cells on apigenin treatment show a significant decrease. A similar study conducted by Ma et al. (2016) noted that rutin increased the ejection fraction (%) and fractional shortening (%) in mice induced with doxorubicin. Moreover, the cardiac histopathology showed that the fibrosis area was significantly decreased compared to mice administered with doxorubicin alone [145]. Interestingly, this is in line with the apoptotic cell (%) that significantly decreased in the treatment of apigenin. Furthermore, the expression of Bcl-2 was significantly increased in the H9c2-treated apigenin in the doxorubicin group [146].

### 3.5. Cyanidin

A study conducted by Sixue et al. showed that purple sweet potato anthocyanin (PSPA) of doses 400, 600, 800 μg/mL significantly increased the cell viability of H9c2 compared to the control group. The NO secretion and TNF-α were significantly decreased in H9c2 treated with DOX 3 μmol/L + PSPA dose 200 and 400 μg/mL. Interestingly, the CK, LDH, and TMAO also significantly decreased in H9c2 which was treated with DOX 3 μmol/L + PSPA dose 200 and 400 μg/mL [147]. Furthermore, the result of the vivo model showed that mice that were treated with PSPA of doses 100 and 200 mg/kg had serum and heart tissue levels of LDH, CK, TNF-α, TMAO that were significantly decreased compared with the doxorubicin group [147]. Additionally, anthocyanin has the ability to reduce the level of pro-inflammatory cytokine, which increases in both H9c2 and mice treated with doxorubicin [147]. This is in line with the result of histological analysis of the heart, which shows that doxorubicin causes myocardial rupture and an excessive amount of inflammatory infiltration. However, mice that were receiving PSPA 200 observed only small inflammatory infiltration. This is in line with a study contributed by [148], which stated that cyanidin-3 glucoside can reduce the toxicity caused by doxorubicin, showing that it increases cell viability and decreases renin-angiotensin-aldosterone (RAA). Anthocyanins also significantly decreases reactive oxygen species such as ROS, O.2, OH., H_2_O_2_, ONOO-, NO, and the MDA as a marker of lipid peroxidation on H9c2 cell-induced doxorubicin [149]. Generally, cyanidin inhibits apoptosis, pro-inflammatory cytokines, ROS production, and lipid peroxidation; however, it elevates cell viability and antioxidant effects.

### 3.6. Hesperidin

Hesperidin is one of the flavonoids subclasses of flavanones, which has been extensively tested for its pharmacological activities both in vitro, in vivo, and in silico. The antioxidant properties of hesperidin are extremely high; this shows that hesperidin has potent scavenging activity with the inhibitory concentration of 10.60 μg/mL [150]. The results of a study conducted by Trivedi et al. show that the hesperetin dose of 100 mg/kg significantly reduced the MDA in rat serum induced with doxorubicin [151]. Additionally, the expression of NF-κB, p38, and caspase on cardiac histopathology by IHC showed a significant decrease in rats receiving hesperidin of 100 mg/kg compared with the group which was only injected with doxorubicin. In addition to hesperidin, a previous study revealed that hesperidin formulated with solid nanoparticles (SLN) decreased the cardiac markers of CK-MB and troponin I on rats treated with doxorubicin [152]. Moreover, the hesperidin-SLN decreased the lipid peroxidation but increased the CAT and SOD levels [152]. A similar study showed that rats induced with doxorubicin and given hesperidin 50 mg/kg (3 times a week) had significantly decreased levels of LDH, CK, NO, MPO, MDA, and Caspase-3, compared with the groups that given only doxorubicin [153].

### 3.7. Chrysin

Chrysin is a flavonoid discovered in honey, mushrooms, and several plants [154]. Currently, several pharmacological activities of chrysin have been tested on cell and rat models. In cardioprotective activity, chrysin depleted the production of ROS, which caused decreases in protein p38, p53, and Nf-κB in cardiac cells [155]. The activation of the p53 cardiac cell stimulated the protein pro-apoptosis Bax and decreased the protein anti-apoptosis Bcl-2. Doxorubicin plays an important role in increasing PTEN and decreasing VEGF, which downregulates the AKT protein that causes apoptosis [154]. Furthermore, a study conducted by Mantawy showed that chrysin reduced pro-inflammatory markers such as Nf-κB, iNOS, COX-2, and TNF-α on rats intoxicated with doxorubicin. Therefore, Nf-κB plays an essential role in the downstream of the inflammatory response which causes pathological changes in the cardiac cell [156].

### 3.8. Naringenin and Narigin

**Figure 3:**
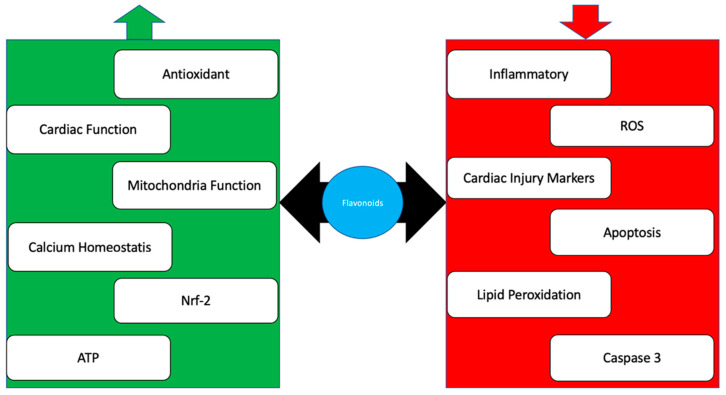
Summary of flavonoids’ role against doxorubicin that increases (green) antioxidant endogen, cardiac function, mitochondria function, calcium homeostasis, nerf 2 expressions, and ATP while reducing (red) inflammatory, ROS, apoptosis, lipid peroxidation, caspase 3 activity.

Naringenin and naringin are flavonoids that belongs to the isoflavonoid subclasses, widely found in grapes and citrus [157]. Furthermore, they have been tested in some pharmacological activities, including against ischemia/reperfusion and cardioprotective activity against doxorubicin [158]. A conducted study showed that naringenin increased antioxidant endogenous activities such as SOD, GPx, GST, Catalase, and GSH [159]. This antioxidant diminishes superoxide anion, superoxide radical, and hydrogen peroxide that increase while being treated with doxorubicin [160]. Moreover, naringenin is known to increase the expression of Nrf-2 as an antioxidant modulator to produce more antioxidants [161]. Subsequently, the cardiac marker injury was also decreased with the co-treatment of naringenin on rats intoxicated with doxorubicin (CK-MB, LDH, AST, and Troponin T) [162]. Meanwhile, the lipid peroxidation was decreased on a rat receiving naringenin. Moreover, inflammation markers such as TNF-α, IL-6, and IL-10 [162] were decreased compared to the previous study. A summary of the mechanism is presented in Figure 3.

**Table 1:** The cardioprotective activity of flavonoids against doxorubicin-induced cardiotoxicity.

Compound	Study Design	Flavonoid Dose	Doxorubicin Dose	Duration	Parameters	References
Luteolin	In vivo (rat)	50 mg/kg 100 mg/kg (P.O 1 week in advance and gastric administration lasted for 5 weeks)	16 mg/kg (Intraperitoneal injection once a week)	5 weeks	↓BNP, ↓CK-MB, ↓MDA, ↓LDH, ↑SOD, ↑Bcl2, ↓Bax, ↑p-AKT, ↓Caspase-3	[48]
Luteolin-7-O-glucoside	In vitro (H9c2)	10 and 20 µM (pre-treated for 24 h)	10 µM (Incubated for 24 h)	48 h	↑Cell viability, ↓apoptosis, ↓ROS, ↑P-PTEN, ↓P-Akt, ↓P-ERK, ↓p-mTOR, ↓p-GSK-3bate	[114]
Luteolin	In vitro (H9c2)	5, 10, 20 µM (pre-treated for 24 h)	20 µM (Incubated for 24 h)	48 h	↑Cell viability, ↓CK, ↓LDH, ↓ROS, ↓ [Ca^2+^]_i_	[118]
Luteolin	In vitro (AMCMs)	1, 10, 50 µM	1 µM	24 h	↓LDH, ↓CK, ↓Apoptosis, ↓ROS, ↑Bcl-2, ↓Bax, ↓Caspase 9, ↑Bnip3, ↑Parkin, ↑Pink1, ↑LC3BII, ↑P62, ↓mTOR, ↑LAMP1, ↑TFEB, ↑Drp1	[117]
Quercetin	In vivo (rat)	10, 25, 50 mg/kg (P.O for 7 weeks)	2 mg/kg (Intraperitoneal once a week until 4 weeks)	7 weeks	↓Blood pressure, ↓HR, ↓LVEDP, ↑coronary flow, ↑+(dp/dt) max, ↑-(dp/dt) max, ↓CK-MB, ↓LDH, ↓Na^+^, ↓K^+^, ↓MDA, ↑GSH, ↑SOD, ↑Catalase, ↑Nrf2	[122]
Quercetin	In vivo (rat)	2 mg/kg (P.O for 7 days)	10 mg/kg (I.V on day 5)	7 days	↓AST, ↓LDH, ↑GSH, ↓BUN, ↓Creatinine, ↓TBRAS	[133]
Quercetin	In vitro (H9c2)	100 µM (pre-treated for 48 h and 96 h)	1 µM	48 h and 96 h	↑CR inhibition, ↓LDH, ↓iron chealting, ↓LPO IC50	[163]
Quercetin	In vitro (H9c2)	50 and 100 µM (Incubated 48 h)	0–16μM (Incubated 48 h)	48 h	↑Cell viability, ↓apoptosis, ↑MMP, ↓ROS, ↑Bmi-1	[128]
	In vivo (Mice)	100 mg/kg (P.O for 10 days)	20 mg/kg (I.P)	48 h	↑LVEF, ↑LVFS, ↓LVEDD, ↓LVESD, ↓LDH, ↓MDA, ↑SOD, ↑Bmi-1	
Quercetin polymeric micelles	In vitro (H9c2)	µM	0.01, 0.1, 1 µM	48 h	↓Caspase 3, ↓caspase 7, ↓ROS, ↓apoptosis	[164]
	In vivo (mice)	3.31 mg/kg (I.V every 3 days for 3 cycle)	6 mg/kg (I.V every 3 days for 3 cycle)	10 days	↓AST, ↓ALT, ↓CK	
Quercetin	In vitro (Neonatal Rat cardiomyocytes)	10,20,40,80 µM (pre-treated for 22 h)	1 µM (incubated 24 h)	48 h (2 h normal condition)	↑Cell viability, ↓LDH, ↓caspase 3, ↓apoptosis, ↑14-3-3γ, ↑MMP, ↑SOD, ↑Catalase, ↑Gpx, ↓MDA, ↑GSH, ↑GSSG	[129]
Quercetin	In vivo (rat)	10 mg/kg (P.O for 6 weeks)	2.5 mg/kg (I.P every 2 days for 2 weeks)	6 weeks	↓CK-MB, ↓LDH, ↓TNF, ↑SOD, ↑CAT, ↓MDA, ↓NO	[131]
Apigenin	In vivo (rat)	25 mg/kg (P.O for 12 days)	2 mg/kg (I.P every 2 days for 12 days)	12 days	↑%EF, ↑%FS, ↓LVIDd, ↓LVISd, ↓LDH, ↓CK-MB, ↓cTn-I, ↓ALT, ↓AST, ↓%Fibrosis, ↓MDA, ↑SOD, ↑Catalase, ↑Bcl-2, ↓Bax, ↓Caspase-3	[137]
Apigenin	In vitro (Murine cardiomyocytes)	20 µM (Incubated for 24 h)	1 µM (incubated for 24 h)	24 h	↑Cell viability, ↓ROS, TBARS, ↑CAT, ↓Carbonyl protein, ↑SOD, ↑GST, ↑GPx, ↑GSH, ↑GR, ↓DNA fragmentation, ↓8-OHdG, ↓Cyt c, ↑Bcl-2, ↓Bax, ↓caspase 3, ↓caspase 9, ↓caspase 8, Apaf-1, FAS, t-Bid, ↓IκBα, ↓NF-κB, PKC-δ, ↓JNK, ↓p38, ↓p53, ↑PI3K, ↑Akt, mTOR, ↓iNOS, ↑HO-1, and ↑Nrf-2	[136]
	In vivo (rat)	100 mg/kg (P.O 7 days)	3 mg/kg (I.P on day 1,3,5)	7 days	↑Total erythrocytes, ↑Haemoglobin, Total leucocytes, ↓Total cholesterol, HDL, TGD, LDH, ↓CK, ↓AST, ↓Troponin I, ↓Troponin T, ↑SOD, ↓Protein carbonyl, ↓ROS, ↓TBARS, ↑CAT, ↑GPx, ↑GST, ↑GSH, ↓8-OHdG, ↑GR, ↓NADPH oxidase, ↓DNA fragmentation, ↓MMP, ↓Cyt C, ↑Bcl-2, ↓Bax, ↓Caspase 3, ↓caspase 9, ↓caspase 8, ↓FAS, ↓t-Bid, ↓IκBα, ↓NF-κB, ↓PKC-δ, ↓JNK, p38, ↓p53, ↑PI3K, ↑Akt, ↑mTOR, ↓iNOS, ↑HO-1, and ↑Nrf-2	
Apigenin	In vivo (mice)	125 and 250 mg/kg (Gastric gavage for 17 days)	3 mg/kg (I.P every 2 days for 16 days)	17 days	↓AST, ↓LDH, ↓CK, ↓Apoptosis, ↓Bax, ↑Bcl-2, ↓Beclin1, ↓LC3, ↑p-mTOR, ↑mTOR, ↑p-AKT, ↑AKT1/2/3, ↑PI3K	[138]
Rutin	In vivo (H9c2)	10, 30, 50, or 70 μM (pre-treated for 1 h)	5μM/pirarubicin (Incubated 24 h)	24 h	↑Cell viability, ↓ROS, ↓Apoptosis, ↓caspase 3, ↓caspase 7, ↓caspase 7, TGF-β1, p-p38 MAPK	[165]
Rutin	In vivo (mice)	100 mg/kg (P.O for 11 weeks)	3 mg/kg (I.P every 2 days for 2 weeks)	11 weeks	↑LVEF, ↑LVFS, ↓%fibrosis,	[166]
	In vitro (cardiomyocytes)	10 μM (pre-treated for 24 h)	1 μM (incubated for 24 h)	48 h	↓Apoptosis, ↑Bcl-2, ↓Caspase 3, ↓P62, ↓LC3BI/II, ↓ATG5	
Rutin	In vivo (mice)	100 μmol/kg (I.P for 5 days)	15 mg/kg (I.P day 1)	5 days	↑GSHpx, ↓MDA, ↓CPK, ↓Total bone marrow, ↓NADPH IC_50_	[146]
Rutin	In vivo (rat)	50 mg/kg (P.O 3 times per week for 3 weeks)	25 mg/kg	3 weeks	↓Total cholestrol, ↑HDL, ↓LDL, ↓CK, ↓LDH, ↓AST, ↑Glutathione, ↑GPx, ↑Glutathione-s-tranasferase, ↓MDA	[167]
Hesperidin	In vivo (rat)	50 mg/kg (Gastric administration 3 times per week for 3 weeks)	4 mg/kg (I.P 3 times per week for 2 weeks)	3 weeks	↓CK, ↓LDH, ↓NO, ↓MPO, ↓MDA, ↑GSH, ↑CAT, ↓Caspase 3	[168]
Anthocyanin	In vitro (HL-1)	0, 5, 25, 125, 250 μM	0, 0.125, 0.25, 0.5, 1, 2, 4 μM	48 h	↑Cell viability, ↓RAS	[148]
Anthocyanin	In vitro (H9c2)	20 and 40 μg/mL (post-treated for 24 h)	1 μM (treated for 6 and 12 h)	36 h	↑Cell viability, ↓apoptosis, ↓CHIP, ↑HSF1, ↓IGF-IIR, ↓caspase 3, p-NFκB, ↑p-Akt, ↑ERα, ↑ERβ	[149]
Chrysin	In vivo (rat)	25 and 50 mg/kg (P.O for 12 days)	15 mg/kg (I.P on day 12)	12 days	↓CK-MB, ↓LDH, ↓MDA, ↓NF-κB, ↓iNOS, ↓COX-2, ↓Bax, ↑Bcl2, ↓TNF-α, COX-2, ↑SOD, ↑CAT, ↓NO, ↓Apoptosis, ↑GSH, ↑Cyc C	[155]
Chrysin	In vivo (rat)	50 mg/kg (P.O 4 times per week for 5 weeks)	5 mg/kg (I.P once a week for 4 weeks)	4 weeks	↓VEGF, ↑AKT, ↑PTEN, ↓NF-κB, ↓Bax, Bcl-2, ↓P53, ↓MAPK, GSH, ↓MDA, ↑CAT, ↑SOD, ↑Gpx, ↑GR	[155]
Hesperidin	In vivo (rat)	25, 50, 100 mg/kg (P.O 5 times per weeks for 5 weeks)	4 mg/kg (I.P once a week for 5 weeks)	5 weeks	↓MDA, ↑GSH, ↓NF-kB, ↓p38, ↓Caspase-3, ↓apoptosis, ↓% demaged cell	[151]
Hesperidin solid nano particle	In vivo (rat)	20 mg/kg (P.O for 7 days)	15 mg/kg (I.P on day 5)	7 days	↓CK-MB, ↓Troponin I, ↓MDA, ↑SOD, ↑CAT, ↓Apoptosis, ↓Caspase 3	[152]
Anthocyanin	In vitro (H9c2)	100–800 μg/mL	3 μmol/L for 12 h	12 h	↓NO, ↓TNF-α, ↓TMAO, ↓LDH, ↓CK	[169]
	In vivo (mice)	100 and 200 mg/kg (P.O for 25 days)	13 mg/kg injected on day 26, 27, and 18	28 days	↓NO, ↓LDH, ↓CK, ↓TNF-α ↓TMAO	
Naringenin	In vivo (rat)	25 mg/kg (P.O for 7 days)	15 mg/kg (I.P on day 7)	7 days	↓LDH, ↓CPK, ↓MDA, ↑SOD, ↑GSH, ↑CAT, ↑GST	[170]
Naringenin	In vivo (rat)	100 mg/kg (P.O for 2 weeks)	15 mg/kg (I.P on day 14)	2 weeks	↓CK-MB, ↓Creatinine, ↓AST, ↓ALT, ↓Urea, ↓LDH, ↓TNF-α, ↓IL-6, ↓IL-1β, ↓TBARS, ↑GSH, ↑CAT, ↑SOD, ↑GST, ↑GPx	[160]
naringenin-7-O-glucoside	In vitro (H9c2)	5, 10, 20, 40, and 80 μM (pre-treated for 24 h)	10 μM (Incubated 24 h)	48 h	↓Cell viability, ↓ROS, ↓LDH, ↓CK, ↑GSH, ↑GPx, ↓ [Ca2+]I	[171]
Naringenin	In vivo (rat)	15 mg/kg (P.O for 30 days)	15 mg/kg (I.P on day 30)	30 days	↑SOD, ↑CAT, ↑GSH	[159]
Naringin	In vivo (rat)	50 and 100 mg/kg (I.P for 14 days)	15 mg/kg (I.P on day 10)	14 days	↑GSH, ↑SOD, ↑CAT, ↓MDA, ↓NADH, ↓Cyt-C,	[161]
Naringin	In vivo (rat)	50 mg/kg (P.O for 10 weeks)	3 mg/kg (I.P on week 1,3,5,7,9)	10 weeks	↓LDH, ↓Troponin T, ↓MDA, ↑CAT, ↑SOD, ↑GPx, ↓TGFβ1, ↓TNF-α, ↓IL-6, ↓IL-10	[162]

BNP: brain natriuretic peptide; CK-MB: creatinine kinase-MB; MDA: malondialdehyde; LDH: lactate dehydrogenase; SOD: superoxide dismutase; Bcl2: B-cell lymphome2; TNF-α: tumor necrosis factor; IL: interleukin; ROS: reactive oxygen species; Cty-c: cytochrome c; GPx: glutathione peroxidase; PTEN; phosphatase and tensin homolog; NO: nitrite oxide; MMP: mitochondria membrane potential; GSH: glutathione; CAT: catalase; NADH: nicotinamide adenine dinucleotide; HDL: high density lipoprotein; LDL: low density lipoprotein; RAS: renin angiotensin aldosterone; iNOS: inducible nitrite oxide; LVEF: left ventricular ejection fraction; LVFS: left ventricular ejection shortening; NF-κB: nuclear factor kappa B; HR: heart rate; 8-OHdG: 8-Oxo-2’-deoxyguanosine; CR: carbonyl reductase; HO-1: heme oxygenase-1; nrf-2: Nuclear factor-erythroid factor 2-related factor 2; AST: aspartate transaminase; ALT: alanine transferase; TGFβ1: transforming growth beta 1; MAPK: mitogen activated protein kinase; COX-2: cyclooxygenase; Bnip3: BCL2 interacting protein 3.

## 4. Conclusions

In conclusion, doxorubicin has multiple mechanisms that cause cardiac toxicity, including decreased antioxidant effects, decreased mitochondrial function, increased lipid peroxidation, and increased inflammatory response. Furthermore, it has been acknowledged that no study elucidates the predominant mechanism. The dietary supplements in flavonoids, such as quercetin, rutin, luteolin, apigenin, hesperidin, anthocyanin, and naringenin, play an essential role in combatting cardiac toxicity by multiple mechanisms which are reducing ROS, lipid peroxidation, mitochondria permeability and the suppress apoptosis (Figure 3). In the future, it is suggested that more mechanism activities of flavonoids against doxorubicin-induced cardiotoxicity are explored.

## Data Availability

Not applicable.
